# Finger-Individuating Exoskeleton System with Non-Contact Leader–Follower Control Strategy

**DOI:** 10.3390/bioengineering11080754

**Published:** 2024-07-25

**Authors:** Zhenyu Sun, Xiaobei Jing, Xinyu Zhang, Biaofeng Shan, Yinlai Jiang, Guanglin Li, Hiroshi Yokoi, Xu Yong

**Affiliations:** 1 CAS Key Laboratory of Human-Machine Intelligence-Synergy Systems, Shenzhen Institute of Advanced Technology (SIAT), Chinese Academy of Sciences (CAS), and the SIAT Branch, Shenzhen Institute of Artificial Intelligence and Robotics for Society, Shenzhen 518055, China; s1842011@edu.cc.uec.ac.jp (Z.S.); xb.jing@siat.ac.cn (X.J.); c2144003@edu.cc.uec.ac.jp (X.Z.); 2 Department of Mechanical Engineering and Intelligent Systems, The University of Electro-Communications (UEC), Tokyo 182-8585, Japan; jiang@hi.mce.uec.ac.jp (Y.J.); yokoi@mce.uec.ac.jp (H.Y.); 3 Joint Doctoral Program for Sustainability Research, The University of Electro-Communications (UEC), Tokyo 182-8585, Japan; 4 Second People’s Hospital of Lanzhou City, and the First School of Clinical Medicine, Lanzhou University, Lanzhou 730000, China; danbf2023@lzu.edu.cn; 5 Shandong Zhongke Advanced Technology Co., Ltd., Jinan 250000, China

**Keywords:** finger-individuating exoskeleton, non-contact, leader–follower control strategy, accurate motions

## Abstract

This paper proposes a novel finger-individuating exoskeleton system with a non-contact leader–follower control strategy that effectively combines motion functionality and individual adaptability. Our solution comprises the following two interactive components: the leader side and the follower side. The leader side processes joint angle information from the healthy hand during motion via a Leap Motion Controller as the system input, providing more flexible and active operations owing to the non-contact manner. Then, as the follower side, the exoskeleton is driven to assist the user’s hand for rehabilitation training according to the input. The exoskeleton mechanism is designed as a universal module that can adapt to various digit sizes and weighs only 40 g. Additionally, the current motion of the exoskeleton is fed back to the system in real time, forming a closed loop to ensure control accuracy. Finally, four experiments validate the design effectiveness and motion performance of the proposed exoskeleton system. The experimental results indicate that our prototype can provide an average force of about 16.5 N for the whole hand during flexing, and the success rate reaches 82.03% in grasping tasks. Importantly, the proposed prototype holds promise for improving rehabilitation outcomes, offering diverse options for different stroke stages or application scenarios.

## 1. Introduction

The motor function of the human hand plays a crucial role in the activities of daily living (ADLs). It is well known that impairments of the hand motor function affect not only quality of life but also lead to negative psychological problems [1,2]. With the aging of the global population intensifying, an increasing number of people have impairments in hand function due to neurological or musculoskeletal diseases, with stroke being the third most common disease [3,4,5]. Additionally, recent research data indicate that more than 12.2 million new stroke patients are diagnosed each year [6,7]. Particularly, in the first week after stroke onset, the proportion of patients with upper limb motor dysfunction is as high as 69% [8]. In general, the earlier the stroke patient receives professional guidance or treatment from the physical and occupational therapists, the more beneficial it will be to recovery [9]. Globally, the average availability of physiotherapists is only 3.6 per 10,000 people. However, in the Asia–Pacific and Western Pacific region, the figure drops to 1.47, ranking second from the bottom [10,11]. In fact, it is often difficult for patients to obtain timely rehabilitation services, resulting in missing the golden recovery period and further causing irreversible disability [12]. Regarding this shortage of the presence of rehabilitation service providers, rehabilitation devices have been suggested to extend therapy capabilities for stroke survivors [13,14]. As an effective approach for assisting patients in recovering hand motor functions and alleviating the shortage of physiotherapists, exoskeleton systems have been extensively studied [15,16].

Currently, various exoskeletons have been developed to enhance impaired hand motor functions, aiming to provide easy operation and wide applicability in rehabilitation [17]. However, achieving the accurate and individual movements of finger joints for the impaired hand remains a significant challenge [18]. Existing exoskeleton designs employ a variety of underactuated transmissions, including tendon-driven mechanisms, linkage mechanisms, and even both. Typically, the tendon-driven mechanism is often utilized in glove-type exoskeletons [6,19,20,21,22,23,24,25,26]. This kind of design usually possesses advantages, such as a light weight and comfort when wearing, owing to the external configuration of the actuation parts and the flexibility of both tendons and gloves. Nevertheless, most of them primarily perform well in assisting continuous movements involving multiple finger joints rather than providing accurate movements for the individual joints. Additionally, the propensity for the glove to deform may sometimes result in a loss of output efficiency and assisting effect in rehabilitation training. Additionally, although exoskeletons with linkage mechanisms [27,28,29,30,31,32,33,34,35] compensate for this deficiency to some extent, their whole systems are generally bulky and difficult to personalize for the different demands of stroke stages or application scenarios. Thus, it is necessary to consider both lightweight and stable transmissions in the exoskeleton design.

On the other hand, the control strategies for existing hand exoskeletons, which provide users with convenient and active operations, are mainly divided into passive and active approaches. Passive control strategies, which typically rely on position and force feedback as the primary control parameters, guide the impaired hand to execute repetitive training tasks based on mechanical structures or preset procedures. For instance, Ref. [25] utilized the deformation characteristics of shape memory alloys to provide actuation forces during training, while [27,28] adopted preset programs to guide the user in rehabilitation. While these designs feature simple structures and easy control, they may compromise the requirements for active consciousness control precision and active operation to some extent, whereas active control strategies utilize sensors and algorithms to acquire real-time information about the user’s movement intention or joint position, thereby enabling more accurate and active assistance in hand rehabilitation. Typically, they use electroencephalography (EEG) and electromyography (EMG) signals [19,30,35,36] as control inputs. However, the acquisition and application of EEG and EMG signals require prior calibration or learning processes, which usually require more complicated control systems, and some patients may not be completely receptive to the contact or implantation of electrodes due to some risks such as rejection or allergy. Some research [20,37,38] indicates that the non-contact sensing method could offer greater practicability and efficiency for motion intention or information acquisition, such as the augmented reality (AR) and Kinect-based motion analysis devices. However, in AR applications, higher hardware performance and optimization algorithms are often required to reduce system latency. Furthermore, the Kinect-based method is more suitable for acquiring and analyzing motion information for large joints rather than accurately tracking the movement of individual finger joints.

**Figure 1:**
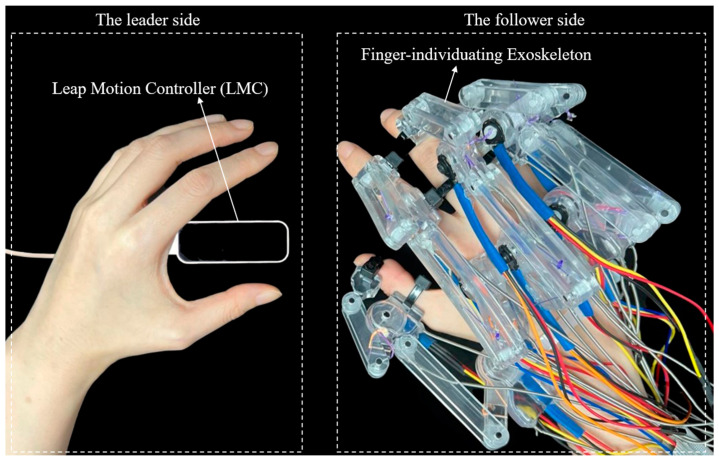
The proposed exoskeleton system applied for the whole hand.

In light of the aforementioned considerations, this study proposes a novel finger-individuating exoskeleton system with a non-contact leader–follower control strategy capable of adapting to various digit sizes. Figure 1 illustrates the system when applied to the whole hand, from the thumb to little finger. Specifically, we utilize a Leap Motion Controller to capture finger joint motions as the control input, enabling rapid and accurate motion tracking of individual joints on the leader side. Subsequently, on the follower side, real-time feedback of the exoskeleton’s current motion is collected to form an interactive loop with the leader side, ensuring control accuracy. Consequently, our solution effectively integrates both motion functionality and individual adaptability, offering diverse options for different stages of stroke recovery or application scenarios.

The rest of this paper is organized as follows: Section 2 details the mechanism of the hand exoskeleton and describes the non-contact leader–follower control strategy. Specifically, it covers the mechanical design, kinematic analysis, and the control strategy utilized to achieve precise and adaptive finger motion. Section 3 presents the experimental results and accompanying discussions, highlighting the effectiveness of the exoskeleton in terms of force output, control accuracy, assistive range of motion, and grasping performance. The experimental procedures, data analysis, and the interpretation of results are thoroughly examined to validate the design and performance of the proposed system. Finally, Section 4 concludes this work by summarizing the key findings, discussing potential applications, and suggesting directions for future research.

## 2. Materials and Methods

### 2.1. Related Work

As a wearable device, hand exoskeleton systems typically utilize the human body’s biomechanical information to drive exoskeleton mechanisms, assisting patients in rehabilitation training to improve or restore the movement function of the affected hand side. So far, a large number of studies related to hand exoskeletons have emerged. Regarding existing hand exoskeleton systems, we have analyzed them from five aspects, as shown in Table 1. The following content discusses these five aspects, respectively, as detailed below.

1. Transmission Method

The transmission method of the exoskeleton mechanism is a crucial factor that directly affects the effectiveness of assisting the movement of the affected hand side. Studies such as [21,22,27,28,29,30,31] have employed various transmission methods, including flexible tendons, rigid links, and a combination of rigidity and flexibility. Currently, the transmission method of flexible tendons is mostly applied in glove-type exoskeletons [21]. Due to the separation of the motor-driven part from the glove part, a significant lightweight effect is achieved. However, glove-type exoskeletons can only achieve coordinated motion assistance for multiple joints of the fingers and cannot provide precise movement assistance for individual joints. Moreover, the soft material of glove-type exoskeletons is prone to deformation and displacement, leading to issues such as a low force transmission efficiency, suboptimal assistance effects, and poor durability. On the other hand, the rigid transmission method based on links [22,27,28], while improving the aforementioned issues of glove-type exoskeletons to some extent, has drawbacks. The inability to separate the motor-driven part from the mechanism can lead to an overall increase in system weight, which is not conducive to prolonged patient rehabilitation training. Additionally, it is challenging to achieve precise movement assistance for individual joints. Concerning the hybrid transmission method, combining rigidity with flexibility, rigid links provide stability and power transmission, while flexible tendons allow for the separation between the motor-driven part and the mechanism, making it easier for patients to use hand exoskeletons. However, the drawback of the hybrid transmission method is that prolonged use of tendons may lead to elastic deformation, affecting the precision of hand exoskeletons.

2. Single-Joint Precision Assistance

The study by [28] conforms to a one-to-one correspondence between a single driving source and a single joint, but it does not impose strict trajectory requirements on other finger joints. Meanwhile, other existing hand exoskeletons generally adopt an underactuated approach, linking multiple joints with fewer driving sources [21,22,26,27,35,39], making it impossible to achieve precise rehabilitation assistance for individual joints. The research by [18] indicates the necessity of precise rehabilitation assistance for individual joints.

3. Actuator

Currently, a variety of drive methods are employed for hand exoskeletons, including linear DC motors, rotary DC motors, servo motors, pneumatic/hydraulic drives, and shape memory alloys. Among them, linear DC motors [29,30] are typically directly coupled at the finger joints, commonly positioned on the dorsal side of the hand, unavoidably increasing the burden on the affected hand, impacting the patient’s usability and comfort. Rotary DC motors [31] have lower costs, are easy to control, but usually cannot offer accurate position control. Servo motors [32] can output high torque and support bidirectional rotation to assist finger flexion and extension, but their cost is relatively high. Pneumatic/hydraulic drives [23,24] have the advantages of adjustable force and speed, but their output is insufficient, the durability is poor, and they cannot provide accurate position feedback. Shape memory alloys [25] exhibit a higher power output ratio in terms of deformation force, but the output motion is lagging, highly nonlinear, and not conducive to control.

4. Wearing Method

Existing hand exoskeletons are commonly secured to the affected hand using various methods, such as wearing on the palm, back, or side of the fingers. Placing the exoskeleton on the palm side [33] prevents users from feeling real objects, which is not conducive to assisting hand movements in daily activities. Wearing the exoskeleton on the side of the fingers [34] results in relatively limited space and is prone to friction and collisions with other fingers. Due to the non-involvement of the back side in gripping interactions, many hand exoskeleton designs choose this wearing method [26]. This method also allows users to maximize the sense of touch during gripping, ensuring interaction with real objects, which is highly beneficial for gradually restoring hand function during the rehabilitation process and in assistive use [40]. Ashish D. Deshpande et al. [41,42] developed a novel index finger exoskeleton based on Bowden cables and series elastic actuators controlled using EMG signals. The mechanical structure exhibits high back drivability and low reflected inertia, making it kinematically and dynamically compatible with human fingers, as well as being lightweight. However, its larger size limits its ability to reliably grasp small objects.

5. Control Strategies

The control strategies for existing hand exoskeletons are mainly divided into passive control strategies and active control strategies. Typically, passive control strategies are suitable for the early stages of rehabilitation, guiding the affected hand’s rehabilitation movements through exoskeleton structures. Among them, position and force feedback are the main control methods adopted in passive strategies. Active control strategies are more applicable in the later stages of rehabilitation, inducing active hand movements by generating forces in cooperation or opposition to the affected hand’s movements. The main approach is force–position hybrid control (admittance control). Additionally, it usually requires the identification of the patient’s intention for movement. Control strategies based on movement intention recognition include EEG brain signal control and sEMG electromyographic signal control. Although the use of EEG brain signals for rehabilitation movements has been demonstrated to be feasible [39], it typically requires electrode implantation for better signal quality, resulting in safety risks such as rejection, inflammation, and surgical failures. On the other hand, methods using non-invasive electrodes to collect EEG signals still face challenges in practical applications due to individual differences, real-time stability, and other factors. Furthermore, the control method utilizing sEMG electromyographic signals [30,35] is also extensively utilized. However, it generally faces limitations in practical applications due to errors caused by muscle fatigue, sweating, the need for calibration during wear, and difficulties in accurately identifying the movement status of individual finger joints.

### 2.2. Design Philosophy of This Research

Based on the above analysis of existing studies on hand exoskeleton systems, we propose our design concepts on the following two aspects:

1. Exoskeleton Design

Based on the anatomical structure of the human hand, a reasonable transmission method and driving mode are designed to provide independent degrees of freedom for fine joints while ensuring that the movement of the hand exoskeleton can accurately mimic and reproduce the natural motion trajectory of the human hand, thereby achieving various daily grasping movements. Furthermore, to guarantee rehabilitation efficacy, the hand exoskeleton must be wearable on the affected hand for extended periods, demanding a structure that is lightweight, adaptable, and comfortable. Therefore, a hybrid rigid–flexible mechanism design was employed. This approach maintains the high rigidity of the linkages while enabling the placement of the driving unit at a distance, aiding in the precise rehabilitation and lightweight nature of the single-joint hand exoskeleton, ensuring effective force transmission and portability.

2. Motion Control Strategy

Since the hand exoskeleton needs to meet the leader–follower symmetrical movement of the user’s bilateral limbs, it should move according to the user’s movements. Thus, besides gathering hand movement information and accurately extracting feature quantities, it must also possess real-time capabilities to achieve optimal bilateral movement effects. Moreover, the stability and robustness of the control strategy are crucial. This is essential not only when the hand exoskeleton aids in the gripping interaction between the human hand and objects but also in addressing sudden external disturbances by ensuring a certain level of safety protection. Thus, this study employs a non-contact control strategy based on the LMC. This strategy demonstrates superior individual adaptability, devoid of rejection reactions, and eliminates the risks associated with allergies or electrode displacement. Furthermore, this non-contact rehabilitation system can effectively reduce the potential risk of disease transmission.

### 2.3. Finger-Individuating Exoskeleton Mechanism

The finger-individuating exoskeleton utilizes a tendon–linkage transmission with a universal structural design, allowing it to adapt to any digit of the human hand. Thus, it could assist both individual and combined finger movements for rehabilitation. This section focuses on the mechanical design, including the kinematics and actuation mechanism.

**Figure 2:**
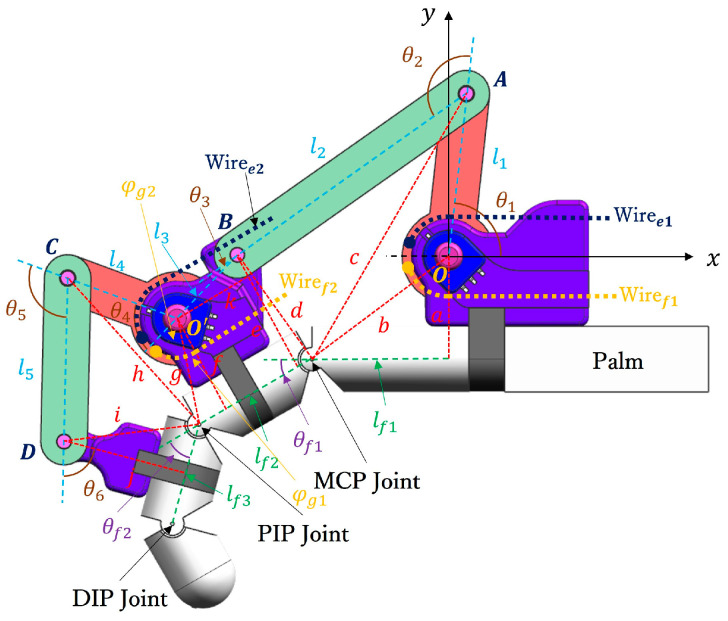
The human–machine coupling model of the exoskeleton worn on the index finger.

**Figure 3:**
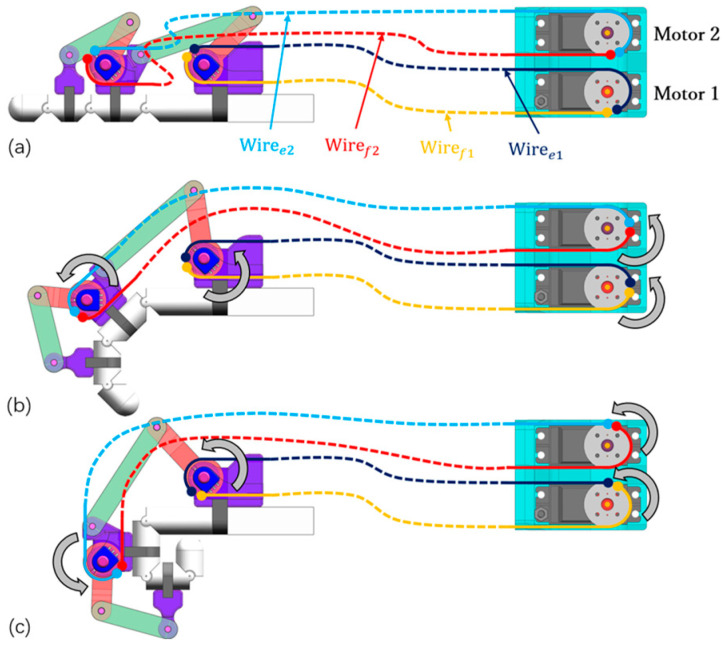
The actuation mechanism: (**a**) full extension; (**b**) medium flexion; (**c**) full flexion.

**Figure 4:**
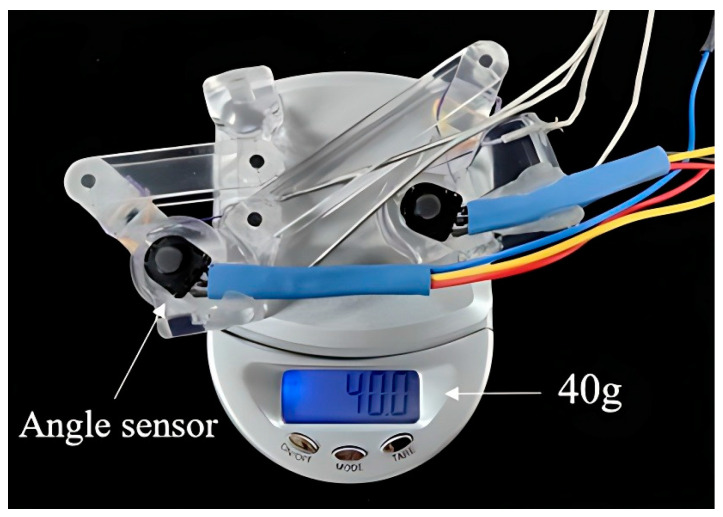
The weight of finger-individuating exoskeleton.

Figure 2 illustrates the human–machine coupling model with the exoskeleton on the index finger. The exoskeleton consists of three bases (purple) and four linkages (*OA*, *AB*, *O*’*C*, and *CD*). Each base frame attaches to the corresponding finger segment with a plastic buckle tie (gray). Two wires, ${Wire}_{e1}$ and ${Wire}_{f1}$, are symmetrically configured at the linkage rotation joint *O*, assisting extension/flexion for the MCP joint. Similarly, ${Wire}_{e1}$ and ${Wire}_{f1}$ are configured at the linkage rotation joint *O*’, assisting extension/flexion for the PIP joint. Figure 3 shows the actuation mechanism based on the human–machine coupling model. Firstly, Figure 3a shows the initial position of the exoskeleton when the index finger is fully extended, where ${Wire}_{e1}$ and ${Wire}_{f1}$ connect to Motor 1, and ${Wire}_{e2}$ and ${Wire}_{f2}$ connect to Motor 2. Secondly, Figure 3b,c demonstrate the flexion motion process. As the two motors rotate counterclockwise, ${Wire}_{f1}$ and ${Wire}_{f2}$ are pulled to rotate the linkages *OA* and *O*’*C*, transferring pushing force to the two distal bases through the linkages *AB* and *CD*, thereby realizing flexion motions of the MCP and PIP joints. Similarly, as the two motors rotate clockwise, ${Wire}_{e1}$ and ${Wire}_{e2}$ achieve extension motions of the MCP and PIP joints, respectively. Additionally, when the exoskeleton is worn on the thumb, it will assist extension/flexion for the MP and IP joints. Thus, the whole-hand exoskeleton possesses 10 degrees of freedom for the whole hand, as shown in Figure 1. In addition, the structure is simple and compact, resulting in a light weight of only 40 g for the finger-individuating exoskeleton, as shown in Figure 4.

Firstly, for the index finger, the kinematic analysis is as follows. Establishing the world coordinate system with the rotation center *O* as the origin, and defining counterclockwise as the positive rotation direction, as shown in Figure 2, $l_{f1}$ represents the distance between the horizontal coordinate projection of *O* and the MCP joint, $l_{f2}$ and $l_{f3}$ represent the lengths of the proximal and intermediate phalanges, respectively, and $\theta_{f1}$ and $\theta_{f2}$ are the rotation angles of the MCP and PIP joints, respectively. The rotation centers of the MCP, PIP, and DIP joints are named sequentially as ${joint}_{1}$, ${joint}_{2}$, and ${joint}_{3}$. Based on the D-H method, the transformation matrix from the origin *O* to the MCP joint can be expressed as follows:

(1) Tjoint1  0=10010−lf10000001001

Similarly, the transformation matrix from the MCP joint to the PIP joint is as follows:

(2) Tjoint2  1=cos⁡θf1−sin⁡θf10−lf2sin⁡θf1cos⁡θf10000100001

The transformation matrix from the PIP joint to the DIP joint is as follows:

(3) Tjoint3  2=cos⁡θf2−sin⁡θf20−lf3sin⁡θf2cos⁡θf20000100001

Therefore, the transformation matrix of the DIP joint relative to the origin O can be described as follows:

(4) Tjoint3  0=∏n=13Tjointn  n−1

Then, for the exoskeleton, $l_{1}$ to $l_{5}$ are defined as the lengths of each linkage from *OA* to *CD*, where $l_{1}$, $l_{2}$, $l_{3}$, $l_{4}$, and $l_{5}$ denote the lengths of linkages *OA*, *AB*, *BO*’, *O*’*C*, and *CD*, respectively, as shown in Figure 2. Additionally, $\theta_{1}$, $\theta_{2}$, $\theta_{3}$, $\theta_{4}$, and $\theta_{5}$ represent the angles between adjacent linkages and base frames, as shown in Figure 2. For example, $\theta_{1}$ is the angle between the linkage *OA* and the base frame fixed on the palm, and $\theta_{2}$ is the angle between the linkages *OA* and *AB*. Then, the linkage rotation joints at *A*, *B*, *O*’, *C,* and *D* are named as ${exo}_{1}$, ${exo}_{2}$, ${exo}_{3}$, ${exo}_{4}$, and ${exo}_{5}$, respectively. Similarly, the transformation matrix from the origin *O* to each linkage rotation joint can be obtained according to the D-H method. For instance, the transformation matrix from the origin *O* to *A* is as follows:

(5) Texo1  0=cos⁡θ1−sin⁡θ10l1sin⁡θ1cos⁡θ10000100001

Similarly, the transformation matrix from *A* to *B* is as follows:

(6) Texo2  1=cos⁡θ2−sin⁡θ20l2sin⁡θ2cos⁡θ20000100001

Thus, the transformation matrix from the origin *O* to *D* can be obtained as follows:

(7) Texo5  0=∏q=15Texoq  q−1

Finally, as shown in Figure 2, the exoskeleton and index finger form a closed linkage mechanism. Therefore, the relation between the finger joints $\theta_{f1}$/$\theta_{f2}$ and the exoskeleton driving angles $\theta_{1}$/$\theta_{4}$ can be calculated by connecting (4) and (7) with trigonometry, as follows:

(8) $$\theta_{f1}={tan}^{-1}\frac{e}{l_{f1}}-\pi +\theta_{1}-{cos}^{-1}\frac{l_{1}^{2}+c^{2}-b^{2}}{2cl_{1}}+{cos}^{-1}\frac{d^{2}+c^{2}-l_{2}^{2}}{2cd}$$

(9) $$\theta_{f2}={tan}^{-1}\frac{j}{l_{f4}}+{cos}^{-1}\frac{k}{l_{3}}-\theta_{4}-{cos}^{-1}\frac{h^{2}+l_{4}^{2}-g^{2}}{2hl_{4}}+{cos}^{-1}\frac{h^{2}+i^{2}-l_{5}^{2}}{2hi}$$

where the geometric parameters from a to j are defined in Figure 2. Specifically, the parameters *b*, *c*, *d*, *g*, *h*, and *i* represent the distances between two adjacent rotation joints. For example, b is the distance between the rotation center O and the MCP joint, and *i* is the distance between the linkage joint *D* and the PIP joint. Additionally, the parameters *a*, *e*, *k*, *f*, and *j* are orthogonal auxiliary lines. For example, *a* is the orthogonal auxiliary line from the rotation center *O* to $l_{f1}$, and *j* is the orthogonal auxiliary line from the linkage joint *D* to $l_{f3}$. In addition, according to the proposed exoskeleton, the geometric parameters from *a* to *j* are constants, so that (8) and (9) can be further simplified as follows:

(10) $$\theta_{f1}=\theta_{1}-acos(\frac{\left(-9.63\right)*\cos\left(\theta_{1}-3.81\right)+28.2}{49.7*\sqrt{1-cos(\theta_{1}-3.81)}})-1.67$$

(11) $$\theta_{f2}=\mathrm{acos}\left(\frac{3.36*(\cos\left(\theta_{4}+0.963\right)-11.5)}{34*\sqrt{1-\cos\left(\theta_{4}+0.963\right)}}\right)-\theta_{4}-1.48$$

The mechanical analysis above provides a theoretical basis for determining both the mechanical and control parameters, which will be elaborated in the following.

### 2.4. Mechanical Parameters

**Figure 5:**
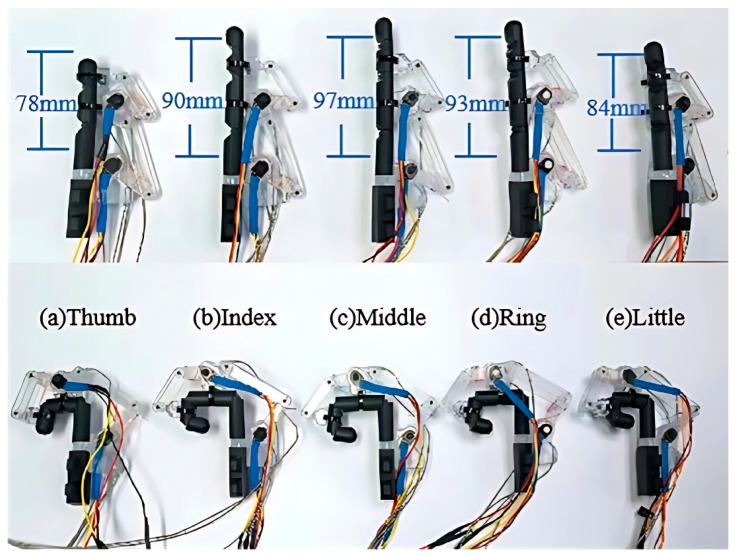
The individual adaptability of the exoskeleton to various digits (3D-printed parts) in full extension/flexion: (**a**) thumb, (**b**) index, (**c**) middle, (**d**) ring, and (**e**) little fingers.

To adapt to the diverse sizes of different fingers, we determined the mechanical parameters of the exoskeleton, including the linkage length and joint rotation range, as detailed in Table 2. Using acrylonitrile butadiene styrene material in transparent color, we implemented a prototype of the exoskeleton through 3D printing. Subsequently, we employed five 3D-printed digits (acrylonitrile butadiene styrene, black color) to illustrate the individual adaptability of the prototype exoskeleton more comprehensively. As depicted in Figure 5, these digits encompass a range of sizes, from the thumb to the index fingers. It is evident that the prototype exoskeleton can well adapt to digits of various sizes, enabling full extension/flexion of the MCP and PIP joints for four fingers, as well as the MP and IP joints for the thumb. Furthermore, the digit lengths range from 58 mm to 93 mm, covering the average size of human digits [43].

**Figure 6:**
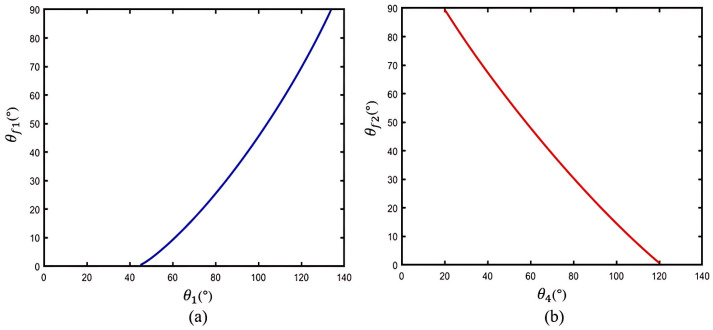
The theoretical relationships based on the human–machine coupling model: (**a**) $\theta_{1}$ and $\theta_{f1}$; (**b**) $\theta_{4}$ and $\theta_{f2}$.

Then, to further verify the human–machine coupling model (see Figure 2), we assume the length parameters of the index finger as detailed in Table 3. By substituting these parameters into (8) and (9), we can establish the theoretical relationships between $\theta_{1}$ and $\theta_{f1}$, as well as between $\theta_{4}$ and $\theta_{f2}$, as depicted in Figure 6.

In Figure 6a, it is illustrated that the MCP joint angle $\theta_{f1}$ increases as the exoskeleton angle $\theta_{1}$ increases, with $\theta_{1}$ flexing to 130° when $\theta_{f1}$ is assisted to reach full flexion at 90°. Then, Figure 6b shows that the PIP joint angle $\theta_{f2}$ increases as the exoskeleton angle $\theta_{4}$ decreases, and $\theta_{4}$ flexes to 20° when $\theta_{f2}$ is assisted to reach full flexion at 90°. The results theoretically indicate that the proposed exoskeleton can assist with extension/flexion of the MCP and PIP joints, while also providing a sufficient rotation range for finger joints during motion.

### 2.5. Non-Contact Leader–Follower Control Strategy

**Figure 7:**
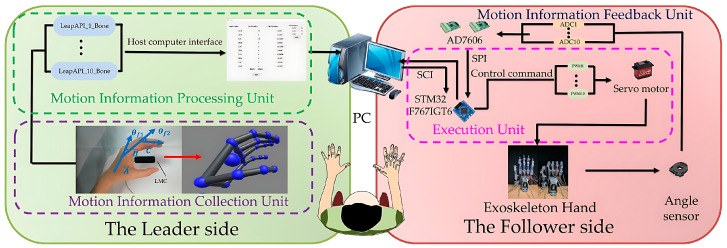
The non-contact leader–follower control strategy.

As depicted in Figure 7, the proposed non-contact leader–follower control strategy comprises the following two interactive components: the leader side and the follower side. The leader side is responsible for collecting and processing joint angle information during movement from the healthy hand, while the follower side executes rehabilitation training for the user’s hand. Moreover, the follower side also provides real-time feedback of the current joint motion on the hand exoskeleton, forming an interactive closed loop with the leader side to ensure control accuracy. Through the collaboration of these two components, our control strategy can offer accurate and active operation for the user. Specifically, the detailed process is as follows.

#### 2.5.1. Leader Side

**Figure 8:**
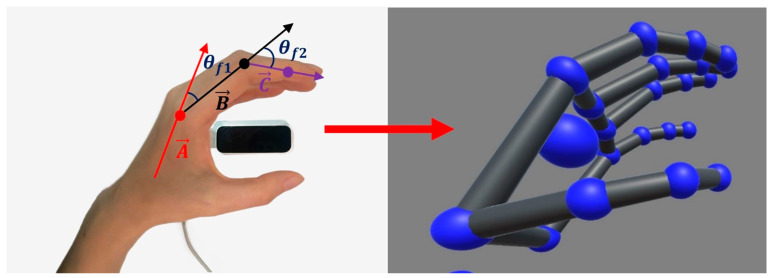
Finger motion acquisition of the healthy hand.

In order to provide an intuitive training experience for the patient, we utilize the movement of the healthy hand as the input for the whole control system, namely, the leader side. A non-contact motion-capture device, the Leap Motion Controller (LMC), is vertically positioned beneath the healthy hand to real-time track the hand position, posture, and speed during motion. The spatial capture range of the LMC is 25–600 mm, with an accuracy of 0.01 mm and a capture speed of 200 FPS. According to the skeletal vector callback function of the LMC, the motion information of the human hand can be obtained, including the angles and direction vectors of all the finger joints, as shown in the skeletal model in Figure 8. Then, since the proposed hand exoskeleton possesses two active degrees of freedom for the MCP and PIP joints of each finger, we can calculate and extract the corresponding joint angles from the skeletal model. Taking the index finger as an example, the vectors of the metacarpal bone, proximal, and intermediate phalanges are defined as $\vec{A}$, $\vec{B}$, and $\vec{C}$, respectively (see Figure 8). Thus, the angles of the MCP and PIP joints, namely, $\theta_{f1}$ and $\theta_{f2}$, can be described as follows:

(12) $$\theta_{f1}=180-{cos}^{-1}(\frac{\vec{A}\cdot \vec{B}}{\vec{\left|A\right|}\cdot \vec{\left|B\right|}})$$

(13) $$\theta_{f2}=180-{cos}^{-1}(\frac{\vec{B}\cdot \vec{C}}{\vec{\left|B\right|}\cdot \vec{\left|C\right|}})$$

**Figure 9:**
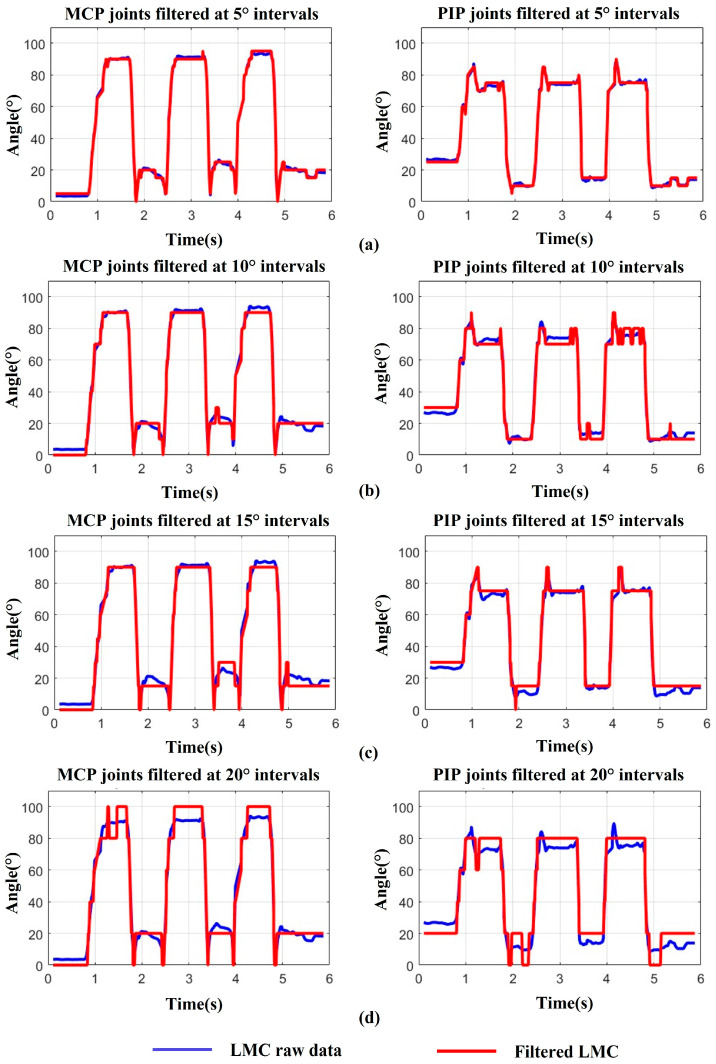
Data smoothing during finger motion: (**a**) MCP, PIP joints filtered at 5° intervals. (**b**) MCP, PIP joints filtered at 10° intervals. (**c**) MCP, PIP joints filtered at 15° intervals. (**d**) MCP, PIP joints filtered at 20° intervals.

In addition, because the LMC has a high sampling frequency of 200 Hz, which may capture the fine tremors of finger joints during movement, we need to smooth the raw data with a proper filter to increase stability and reduce mis-operations as the system input. For data processing, we conducted three trials of full flexion and extension with the index finger on a healthy subject’s right hand, while also recording the angle changes in the MCP and PIP joints. Then, we smoothed the raw data at interval angles of 5°, from 5° to 20°. The results are depicted in Figure 9, where the blue curve represents the recorded data from the LMC, while the red curve illustrates the filtered data corresponding to the filter angles of 5°, 10°, 15°, and 20°, respectively. It can be observed that the filter angle of 20° seems too large, leading to excessive feature loss which the filtered data cannot fit will with the raw data (Figure 9d). Although, with the filter angles of 10° and 15°, the curves fit the raw data much better, error feature spikes on the PIP joint and over filter fine angle changes tend to appear (Figure 9b,c). Only when the filter angle is reduced to 5° does the filtered curve not only fit the raw data very well without significant feature loss, but also becomes much more stable and smoother (Figure 9a). As a consequence, we chose the filter angle of 5° to smooth the raw data during hand movement.

#### 2.5.2. Follower Side

**Figure 10:**
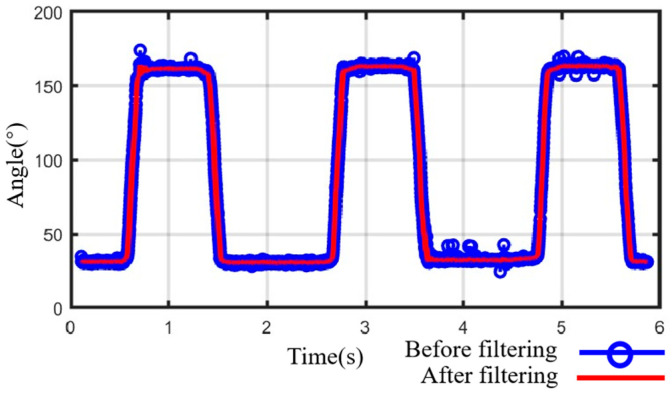
Data processing with median average filtering.

The exoskeleton will follow the leader side to assist the user’s hand in rehabilitation training, known as the follower side. To accurately replicate these motions, two angle sensors (RDC501051A, with an effective electrical range: 333.3°; ALPS ALPINE Co., Ltd., Tokyo, Japan) were installed on the exoskeleton’s rotation centers to provide real-time angle feedback (see Figure 7). The AD7606 processes the angles from sensors during high-frequency sampling. We utilized the median average filtering method to reduce the errors and minimize the impulse interference caused by external impacts, such as grasping objects. Figure 10 displays the results after median average filtering, with the blue curve representing the raw angle data from three full-extension/flexion tests of a single exoskeleton joint, and the red curve illustrating the filtered angle data, which is smoother and more stable.

For the execution unit in Figure 7, the hand exoskeleton’s motion, pulled by tendons, are equipped with ten angle sensors, mentioned above, providing feedback data for finger joint motion. Then, filtered data are sent to the host computer. The host computer processes and fuses the data, then sends them to the execution unit. The execution unit drives Power HD servos (LF-20MG, stall torque 2 N·m, Power HD PQ12-63-6-R, Co., Ltd., Ningbo, China) using PWM signals to move the hand exoskeleton.

To minimize the error between the input and output positions of the system, the position-type PID control method is employed. This method processes the LMC data on the lead side and the angle sensor data on the follow side. Specifically, three parameters, $K_{P}$, $K_{I}$, and $K_{D}$, are used to adjust the deviation between the input and output, as follows:

(14) $$u(k)=K_{p}e(k)+K_{I}\sum_{i=0}e(i)+K_{D}[e(k)-e(k-1)]$$

where *u*(*k*) represents the current output control quantity of the controller, while *e*(*k*) denotes the current deviation signal of the system, equal to the difference between the input and output quantities. $K_{P}$ refers to the proportional coefficient, $K_{I}$ denotes the integral time constant, and $K_{D}$ represents the differential time constant.

To validate the control method, we instructed a healthy subject to operate the exoskeleton using his left finger. The exoskeleton was worn on a 3D-printed finger (see Figure 5b), and the subject’s finger extension/flexion movements were used to drive its MCP and PIP joints to perform the same motions. A total of three extension/flexion tasks were conducted. Throughout the entire process, the MCP and PIP joint angles of the subject were collected using the LMC, while the exoskeleton joint angles were collected using the angle sensor. Additionally, for better comparison, we utilized (8) and (9) to convert the angle sensor data into MCP and PIP joint angles for the 3D-printed index finger.

**Figure 11:**
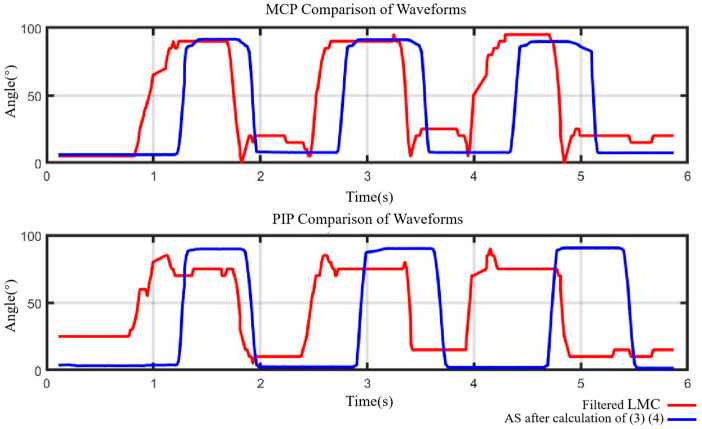
Joint data error comparation of MCP and PIP joints.

As illustrated in Figure 11, the red curve represents the joint angle obtained from the filtered LMC data, while the blue curve represents the joint angle obtained from the angle sensor data (calculated by (8) and (9)). It is evident that the exoskeleton effectively assists the 3D-printed finger joints to perform extension/flexion tasks, exhibiting an average delay of 0.336 s for the MCP joint and 0.565 s for the PIP joint. The delays may be attributed to the differing transmission rates of the LMC and angle sensors. Nonetheless, the average time for the MCP joint to complete one extension/flexion task by the exoskeleton was only 0.814 s, and for the PIP joint, it was only 0.837 s. These time durations are significantly shorter than the 20 s suggested by physiotherapists for a finger joint in a sequence of extension/flexion [44]. Thus, the proposed control method can offer efficient and accurate extension/flexion assistance in rehabilitation training for finger joints.

#### 2.5.3. Control Flow Chart

**Figure 12:**
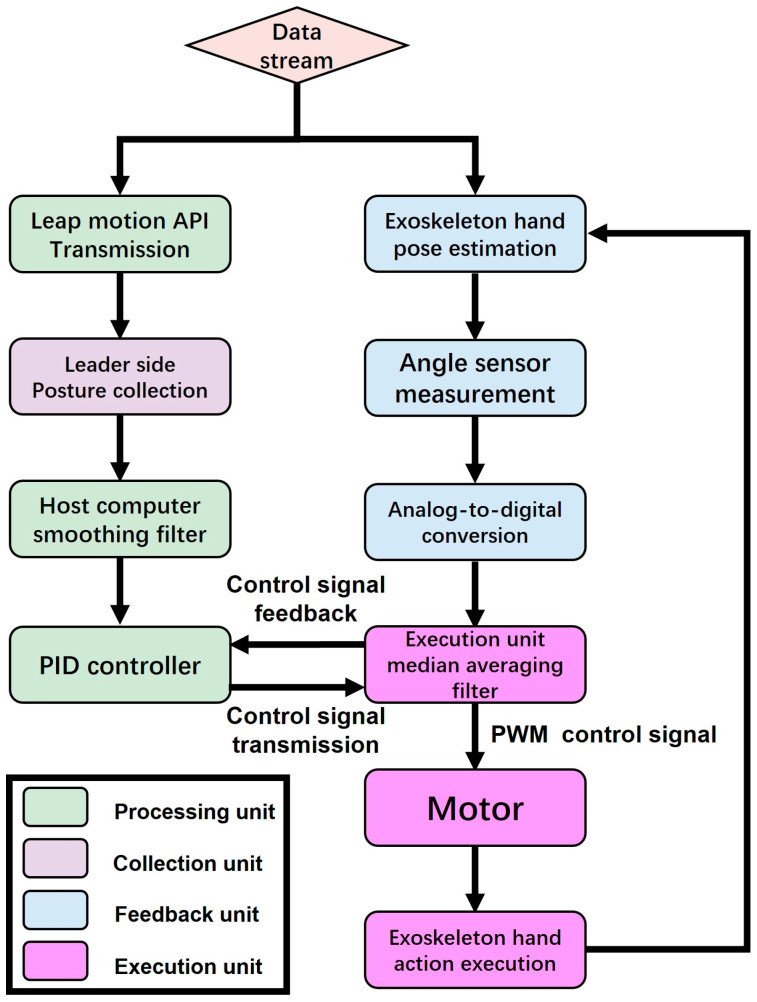
Control flow chart.

The flow chart (Figure 12) illustrates the data stream processing of a hand exoskeleton control system, divided into the following four parts: the processing unit, collection unit, feedback unit, and execution unit. Initially, the data stream enters the system and splits into the following two branches: one branch transmits posture data via the Leap Motion Controller API, while the other estimates the exoskeleton hand posture. In the Leap Motion Controller API transmission branch, the posture data are collected from the leader side and input into the host computer’s smoothing filter. In the exoskeleton hand posture estimation branch, the angle sensor measures the exoskeleton hand’s posture angles and converts the analog signals to digital signals via analog-to-digital conversion. These digital signals are sent to the execution unit, providing more stable feedback signals. Subsequently, the host computer inputs both the smoothed Leap Motion Controller data and the median-averaged angle sensor data into the PID controller simultaneously. The PID controller generates control signals and sends these signals to the execution unit. The execution unit transmits the control signals to the motor in the form of PWM signals. The motor receives the signals and executes the motion of the exoskeleton hand. Meanwhile, the motion of the exoskeleton hand is monitored in real-time by the angle sensors, continuously adjusting to ensure the accuracy and consistency of the motion.

## 3. Results

### 3.1. Experiment

We conducted four experiments to verify the proposed exoskeleton system, including the output force and control accuracy for a single finger, as well as the assistive range of motion and grasping performance for the whole hand.

#### 3.1.1. Flow Chart of the Experiments

**Figure 13:**
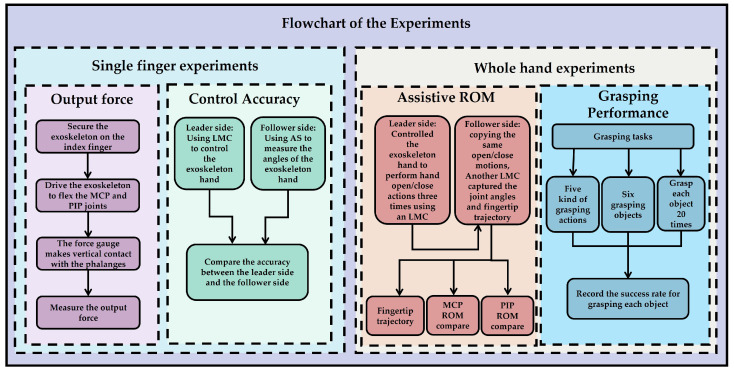
Flow chart of the experiments.

Figure 13 systematically illustrates the design and execution process of the experiment, which is divided into the following two parts: single-finger experiments and whole-hand experiments. The single-finger experiments include an output force experiment and a control accuracy experiment. In the output force experiment, the exoskeleton is fixed on the index finger, driving its flexion at the MCP and PIP joints, with a dynamometer in direct contact with the phalanx to measure the output force. In the control accuracy experiment, the master side uses a Leap Motion Controller (LMC) to control the exoskeleton hand, while the slave side uses an angle sensor to measure the angle of the exoskeleton hand, comparing the angle accuracy between the master and slave sides. The whole-hand experiments include an assisted range of motion (ROM) experiment and a grasping performance experiment. In the assisted ROM experiment, the leader side uses the LMC to control the exoskeleton hand to perform three opening and closing movements, which are replicated by the follower side. Another LMC measures the joint angles and fingertip trajectory, comparing the ROM of the MCP and PIP joints. In the grasping performance experiment, the experiment includes five grasping movements and six grasping objects. Each object is grasped 20 times, and the success rate for each object is recorded. These steps aim to verify the design effectiveness and motion performance of the exoskeleton system, ensuring its practicality and reliability in rehabilitation training.

#### 3.1.2. Experiments for a Single Finger

For better measurement, we conducted experiments on the output force and control accuracy based on the 3D-printed exoskeleton prototype and index finger (see Figure 5b). The experimental process and results are as follows.

1. Output Force

**Figure 14:**
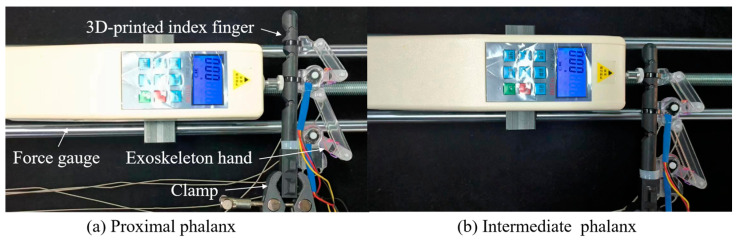
The initial experimental setup.

Figure 14 illustrates the initial experimental setup for the proximal and intermediate phalanges, respectively. First, secure the exoskeleton onto the index finger using adjustable plastic straps in the fully extended position. Secondly, clamp the index finger with a vise and position it horizontally on a table. Next, employ two servo motors to drive the exoskeleton joints, flexing the MCP and PIP joints, respectively. Then, use a force gauge to measure the output force on the proximal and intermediate phalanges. Meanwhile, utilize a goniometer to measure the joint angle of the MCP (see Figure 14a) and PIP (see Figure 14b) joints during flexion, respectively.

As an example of the experimental procedure for measuring the force on the proximal phalanx, the specific steps are as follows: (1). Drive the exoskeleton to flex the MCP joint from the initial position to the full flexion position by the servo motor, i.e., from 0° to 90°. (2). At approximately every 15° of flexion, adjust the force gauge to make vertical contact with the proximal phalanx to measure the output force.

**Figure 15:**
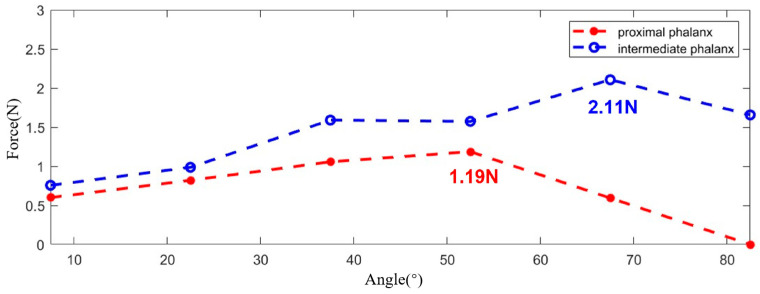
The output force experiment results.

For the proximal phalanx, five measurements were obtained during the entire trial. The measurement values are indicated by red circles in Figure 15. Similarly, the same measurements were conducted for the intermediate phalanx. It is noteworthy that, for better comparison, measurements were taken while maintaining the PIP joint at angles identical to those of the MCP joints. The measured values are marked with blue circles in Figure 15.

Based on the experimental results, it can be observed that the maximum output force is approximately 3.3 N. Further calculations indicate that, when the exoskeleton is worn on all five digits, the maximum output force for the entire hand can reach up to 16.5 N, which falls within the appropriate range for flexion force [45,46].

Table 4 compares the parameters of various exoskeleton hand designs from previous research, focusing on the weight of the devices and the output forces they generate. The Exo-Glove weighs 250 g and produces forces of 3.59 N for the index finger and 1.43 N for the thumb. The HANDEXOS, at 114.9 g, outputs 40 N, while the Maestro, weighing 57 g, provides 12.5 N. K. Y. Tong et al.’s design weighs 500 g and generates 23 N for the whole hand. M. Decker et al.’s model, at 454 g, and the SAFE device, at 430 g, both produce 10 N of force. The exoskeleton hand proposed in this article stands out with its minimal weight of 40 g and an output force of 3.3 N. This comparison highlights the trade-offs between weight and functionality, with the proposed design offering a balance that prioritizes mobility and ease of use.

2. Control Accuracy

**Figure 16:**
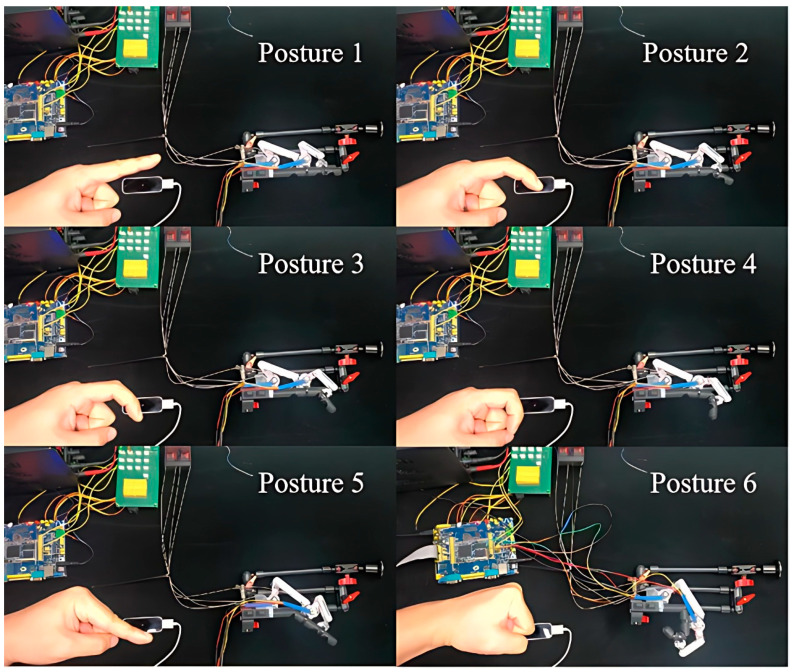
Control accuracy experiment: snapshots and results.

Then, to investigate the control accuracy of the proposed non-contact leader–follower strategy (see Section 2.5), we conducted the following experiments. On the leader side, we employed a healthy subject (32 years old, male) and instructed him to flex his index finger of the left hand upon the LMC. While, on the follower side, we used the same 3D-printed prototype of the exoskeleton and index finger (see Figure 5b). The subject was instructed to flex his MCP or PIP joints, and arbitrarily stop at a position. During this process, the exoskeleton drove the 3D-printed index finger to flex along with the subject’s finger. Furthermore, the angle of the subject’s finger joint (MCP and PIP) was collected by the LMC, while the joint angle of the exoskeleton was also collected by the AS, respectively. Figure 16 shows six scenes measuring arbitrary finger postures during the experiment. And, to better compare the human finger joint angle with that of the 3D-printed index finger, we converted the joint angle measured by the AS to the finger joint angle on the 3D-printed index finger based on (8) and (9). The result is shown in Table 5.

Firstly, from Figure 16, it is clear to see that the exoskeleton follows well the movements of the human finger, assisting the 3D-printed index finger to execute various motions during flexion. Secondly, Table 5 shows that the proposed exoskeleton could assist the MCP and PIP joints to execute individual and combined motions for a single finger. In addition, it can be seen from Table 5 that the instantaneous absolute errors at the MCP and PIP joints are at least 0.99° and at most 5.45°, which proves that the proposed control system has a relatively high precision in actual operation.

#### 3.1.3. Experiments for the Whole Hand

As mentioned in Section 2, the proposed finger-individuating exoskeleton is suitable for any digit of the human hand. Thus, we conducted the following two experiments to further validate the function and performance of the proposed exoskeleton system when applied to the whole hand. The first experiment aimed to test the assistive ROM when worn on all five digits of the hand, as this is a primary focus for physical therapists during rehabilitation and commonly serves as a general indicator for evaluating hand function. The second experiment aimed to verify the grasping performance of the whole hand, as grasping is the most frequent activity in the activities of daily living [47,48].

1. Assistive ROM

In this experiment, the healthy subject was instructed to wear the exoskeleton on his right hand, spanning from the thumb to the little finger. The subject then placed his left hand upon the LMC to perform hand open/close tasks three times, thereby controlling the exoskeleton to assist the right hand in copying the same open/close motions. Throughout this procedure, another LMC was positioned under the right hand to capture the angles of the MCP and PIP joints, as well as the fingertip trajectory.

**Figure 17:**
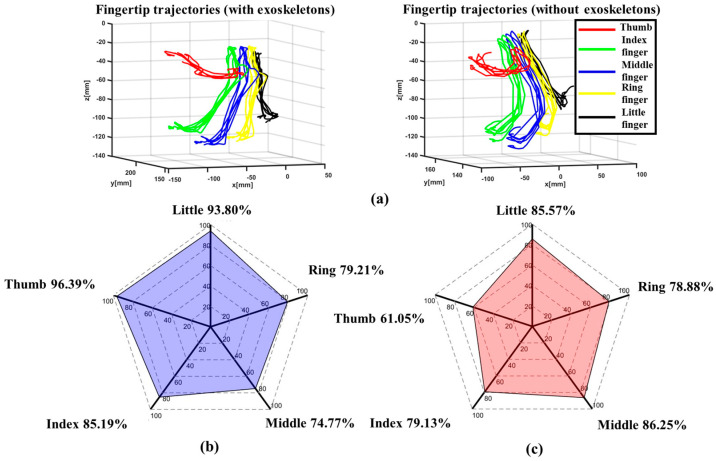
The experimental results: (**a**) trajectories of the fingertip, (**b**) the ROM of the MP/MCP joints in an exoskeleton hand (indicated by the blue shading)/the ROM of the MP/MCP joints in a healthy human hand, and (**c**) the ROM of the IP/PIP joints in an exoskeleton hand (indicated by the red shading)/the ROM of the IP/PIP joints in a healthy human hand.

Figure 17a shows the average fingertip trajectory both with and without the exoskeletons, which are very similar to each other. This indicates that the proposed exoskeleton can assist finger joints in motion in a natural manner similar to the human hand. The average assistive ROM of the MCP and PIP joints of each finger with the exoskeleton is depicted by blue histograms in Figure 17, while the ROM of healthy human finger joints without the exoskeleton is represented by red histograms in the same figure [49]. Compared with the healthy human hand’s ROM, it is evident that the assistive ROM by the exoskeleton for the MP/MCP joints ranges from at least 74.77% to at most 96.39% (see Figure 17b), while, for the IP/PIP joints, the percentages range from at least 61.05% to at most 85.57% (see Figure 17c). Therefore, compared with the human hand, it is indicated that the average assistive ROM provided by the proposed exoskeleton for the whole hand is as high as 82.03%.

2. Grasping Performance

**Figure 18:**
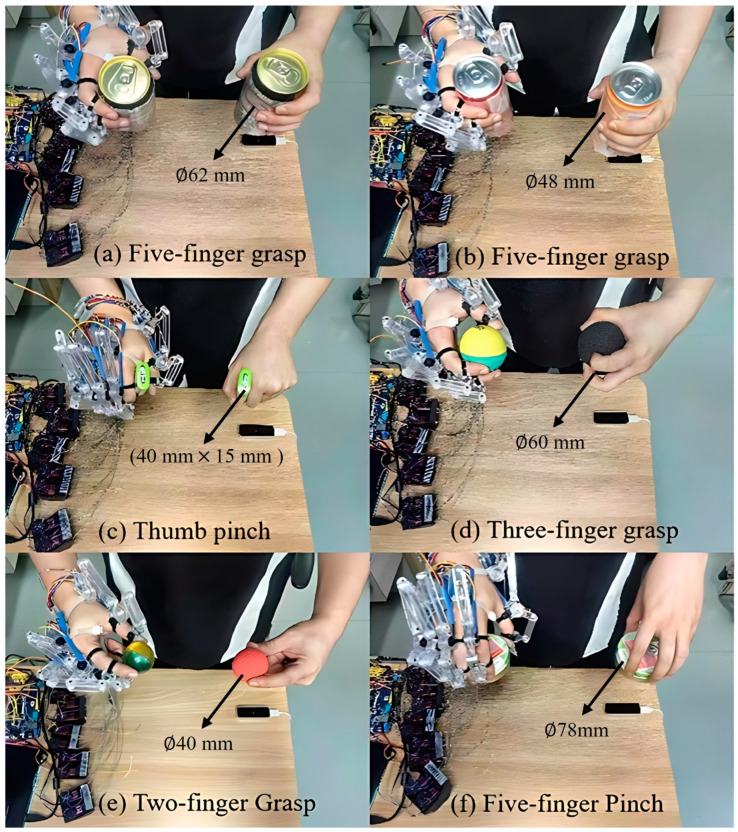
Grasping tasks: (**a**) five-digit grasp of a large soda can, (**b**) five-digit grasp of a medium soda can, (**c**) thumb pinch of a candy box, (**d**) three-digit grasp of a large ball, (**e**) three-digit grasp of a small ball, and (**f**) five-digit pinch of a round box.

**Figure 19:**
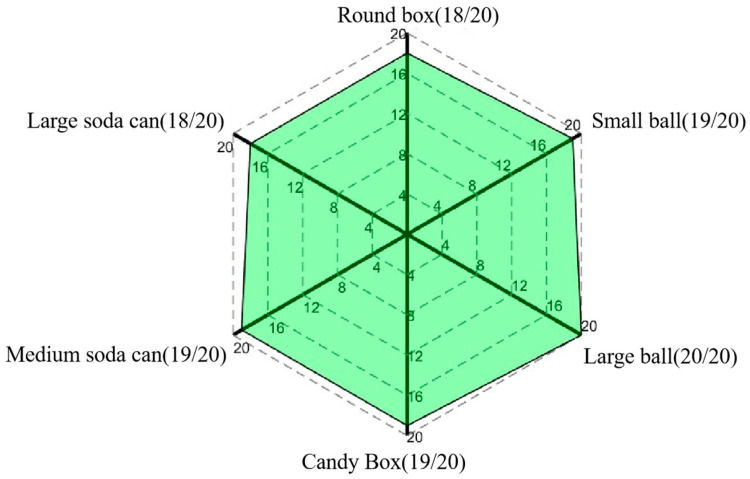
Success rate of each object.

In order to evaluate the grasping performance of the proposed exoskeleton, we instructed the healthy subject to operate the exoskeleton system by his left hand, assisting his right hand to perform grasping tasks with different objects. As shown in Figure 18, five kinds of grasping tasks were conducted, including the five-finger power grasps (see Figure 18a,b), the thumb pinch (see Figure 18c), the three-finger grasp (see Figure 18d), the two-finger pinch (see Figure 18e), and the five-finger pinch (see Figure 18f). Several everyday objects were employed, including a large soda can (62 mm in diameter and 122 mm in height, as shown in Figure 18a), a medium soda can (48 mm in diameter and 145 mm in height, as shown in Figure 18b), a candy box (40 mm in length, 15 mm in width, and 60 mm in height, as shown in Figure 18c), a large ball (60 mm in diameter, as shown in Figure 18d), a small ball (40 mm in diameter, as shown in Figure 18e), and a round box (78 mm in diameter and 25 mm in height, as shown in Figure 18f). To ensure the accuracy of the experiment and to prevent interference from the hands of other experiment participants entering the measuring range of the LMC, a longer gripper was utilized to deliver the object to the subject. For each object, the subject was instructed to grasp 20 times while, at the same time, the gripping success rate was recorded, as shown in Figure 19.

The experimental results clearly indicate that the proposed exoskeleton system can achieve high success rates in grasping tasks with various everyday objects. Specifically, as shown in Figure 19, for spherical objects (balls), a flat object (the candy box), and a cylindrical object (the medium soda can), the number of successful grasps reached 19 to 20 times, resulting in a success rate of more than 95%, while the number of successful grasps was reduced to 18 times for the bigger spherical and disk-shaped objects, such as the large soda can and round box. This may be due to the fact that grasping objects with larger diameters make it harder for the digits to wrap around the object contour, leading to slippage while grasping. Nevertheless, the success rate for all of the trials remained consistently above 90%, thus providing full validation of the high grasping performance and stability of the proposed exoskeleton system.

To summarize, all of the following experimental results clearly demonstrate the efficacy of the proposed exoskeleton system:

- Average Generated Force: The system generates an average force of 16.5 N across all five fingers, which is optimal for hand flexion and rehabilitation exercises.
- Control Accuracy: The exoskeleton replicates the movements of a healthy hand with minimal error margins, ensuring reliable assistance for patients.
- Assistive Range of Motion (ROM): The assistive ROM for the MCP and PIP joints averaged 82.03% of the natural human hand’s ROM, supporting extensive finger movements.
- Grasping Performance: The exoskeleton achieved a success rate consistently above 90% in grasping tasks, even with varying object shapes and sizes.

These results highlight the exoskeleton system’s potential to enhance hand function recovery, providing robust, accurate, and adaptive support for rehabilitation.

## 4. Conclusions

This study presents a novel exoskeleton system tailored for precise motion control and adaptability in rehabilitation. The non-contact leader–follower control, alongside a tendon–linkage mechanism, allows the device to fit any finger and support active motions for the MP/MCP and IP/PIP joints. The experiments with both individual fingers and whole hands reveal that the exoskeleton delivers an average flexion force of 16.5 N and provides a range of motion up to 82.03% of that of a healthy hand. The system also demonstrated superior performance and stability in grasping tasks, maintaining success rates above 90%. The design’s versatility shows promise for various applications across different stages of stroke recovery, potentially enabling a physiotherapist to manage multiple patients simultaneously. Future developments will focus on expanding the degrees of freedom in finger joints to enhance the functionality and performance.

## Figures and Tables

**Table 1:** Related work.

Name	Transmission Mode	Single-Joint Precision Assistance	Actuator	Wearing Method	Control Mode
Popov, D. et al. [21]	Tendon-driven glove	N	DC motor	Dorsal side	Position control
SAFE [27]	Linkage	N	DC motor	Dorsal side	Admittance control
Fiorilla, A.E et al. [28]	Linkage	Y	DC motor	Dorsal side	Position control
Decker, M. et al. [22]	Linkage glove	N	DC motor	Dorsal side	Force feedback control
Sarac, M. et al. [29]	Linkage	N	Linear actuator	Dorsal side	Position control
Tong, K. et al. [30]	Linkage	N	Linear actuator	Dorsal side	EMG control
HANDEX-OS [31]	Tendon-linkage	N	DC motor	Dorsal side	Position control
Allotta, B. et al. [32]	Linkage	N	Servo motor	Dorsal side	Position control
Exo-Glove PM [23]	Glove	N	Pneumatic actuator	Dorsal side	----
Ryu, D. et al. [24]	Glove	N	Hydraulic actuator	Dorsal side	Force feedback control
Kobayashi, F. et al. [25]	Linkage glove	N	Shape memory alloy	Dorsal side	Force feedback control
Rutgers Master II [33]	Linkage	N	Pneumatic actuator	Palmar side	Force–position hybrid control
Cortese, M. et al. [34]	Tendon-driven linkage	N	DC motor	Lateral side	Speed control
Wang, J. et al. [26]	Glove	N	Pneumatic actuator	Dorsal side	Force feedback control
Araujo, R.S. et al. [39]	Linkage glove	N	DC motor	Dorsal side	EEG control
BRAVO Hand [35]	Linkage	N	DC motor	Dorsal side	EMG control

**Table 2:** Parameters of the exoskeleton.

Linkage Length				
*l*_1_	*l*_2_	*l*_3_	*l*_4_	*l*_5_
35 mm	60 mm	18.7647 mm	25 mm	35 mm
Joint Rotation Range				
*θ*_1_			*θ*_4_	
0°–225°			120°–20°	

**Table 3:** Length parameters of the index finger.

$l_{f1}$	$l_{f2}$	$l_{f3}$
27.6 mm	28 mm	22 mm

**Table 4:** Comparison of output forces.

Name	Weight (g)	Output Forces (N)
Exo-Glove [23]	Whole hand, 250 g	Index, 3.59 N; Thumb, 1.43 N
HANDEXOS [31]	114.9 g	40 N
Maestro [41,42]	57 g	12.5 N
K. Y, Tong. et al. [30]	Whole hand, 500 g	Whole hand, 23 N
M. Decker. et al. [22]	Whole hand, 454 g	Whole hand, 10 N
SAFE [27]	Whole hand, 430 g	Maximum force, 10 N
The proposed exoskeleton hand	40 g	3.3 N

**Table 5:** Parameters of accuracy comparison.

	MCP Joint		PIP Joint	
	LMC	3D-Printed Finger	LMC	3D-Printed Finger
Posture 1	5°	5.99°	0°	3.38°
Posture 2	0°	5.96°	25°	23.28°
Posture 3	5°	6.02°	55°	60.47°
Posture 4	0°	5.96°	75°	81.45°
Posture 5	30°	32.35°	0°	3.37°
Posture 6	90°	91.25°	75°	81.42°

## Data Availability

The data that support the findings of this study are available on request from the corresponding author. The data are not publicly available due to privacy or ethical restrictions.
